# Comparative Evaluation of Puffing Effects on Physicochemical and Volatile Profiles of Brown and Refined Rice

**DOI:** 10.3390/foods14162812

**Published:** 2025-08-13

**Authors:** Xiaomei Liu, Yi Zhang, Kai Zhu, Fan Xie, Haoyu Si, Songheng Wu, Bingjie Chen, Qi Zheng, Xiao Wang, Yong Zhao, Yongjin Qiao

**Affiliations:** 1 College of Food Sciences and Technology, Shanghai Ocean University, Shanghai 201306, China; l2961852572@163.com (X.L.); sihaoyu1023@126.com (H.S.); 2 Crop Breeding and Cultivation Research Institute, Shanghai Academy of Agricultural Sciences, Shanghai 201403, China; zhangyi@saas.sh.cn (Y.Z.); zhukai2000hnau@163.com (K.Z.); wsh_magnus@163.com (S.W.); chenbingjie0204@126.com (B.C.); wangxiao.0127@163.com (X.W.); 3 School of Health Science and Engineering, University of Shanghai for Science and Technology, Shanghai 200093, China; xiefan246141@163.com; 4 Shanghai Shuneng Irradiation Technology Co., Ltd., Shanghai 201401, China; zhengqi@saas.sh.cn

**Keywords:** puffing technology, rice, physicochemical properties, flavor compounds

## Abstract

Rice has excellent nutritional quality as a dietary food and is easily puffed. The aim of this study was to investigate the effects of puffing technology on the physicochemical parameters, structure properties and volatile components of brown rice (BR) and refined rice (RR). XRD and FT-IR spectroscopic data demonstrated that puffing under high temperature and pressure conditions triggered starch gelatinization, concurrently reducing starch crystallinity and inducing a V-type polymorphic structure. In addition, it substantially weakened hydrogen bonding networks in rice flour. In detail, 136 volatile compounds of raw and puffed rice were analyzed by HS-SPME-GC-MS, and the results showed that aldehydes, ketones, and pyrazines were the main volatile aroma compounds after puffing. By correlation analysis, benzaldehyde, 2-octenal, 2-methoxy-phenol, and furfural were identified as key contributors. The volatile components, especially ketones and alcohols, were higher in the BR as compared to those in the RR, with a significant difference observed between the two (*p* < 0.05). Combined with sensory evaluation, 1212CH was found to have a high score (17.63). These results could provide a theoretical basis for understanding the effect of puffing on rice flour and the volatile components of puffed products.

## 1. Introduction

Rice (*Oryza sativa* L.), serving as a staple food for approximately half of the global population, supplies the daily energy requirements of billions worldwide. It is mainly found in parts of Africa, Asia, and Latin America, with China leading in the most significant rice production [1,2]. Brown rice (BR) is derived by hulling rice grains, retaining the endosperm, embryo, and bran layers [3]. The rice bran layer is rich in protein, fat, and various vitamins. BR is associated with hypoglycemic, anti-cancer, anti-inflammatory, and anti-aging properties [4]. However, it is often considered to have a rough taste due to its high-fiber bran layer, leading to low consumer acceptance and limiting its consumption and promotion [5]. Therefore, in industrial production, researchers are using modern processing techniques to treat the bran of BR, including consumption or processing into other forms by extruding, fermenting, puffing, flattening, bursting, etc., to produce noodles, puffed rice, flattened rice or popped rice as a way of improving the bad taste of BR [6]. Currently, puffing technology has attracted the attention of researchers due to its characterizations of crispy texture and convenient consumption.

Puffing rice (PR) involves subjecting rice grains to high pressure and temperature, leading to a rapid increase in internal moisture temperature and subsequent phase change that significantly elevates the internal pressure [7]. The sudden release of pressure results in instantaneous water evaporation within the granules, leading to granule swelling and porosity. This process alters the physical characteristics of rice by increasing volume, reducing density, and inducing molecular changes, particularly in the starch structure [8]. Kuo et al. [9] found that puffing destroyed starch granules, decreased starch crystallinity, and enhanced gelatinization and surface area. Mir et al. [10] suggested that puffing destroyed the structure of BR, changed the crystallinity of starch, and formed a porous structure. In addition, PR is an oil-free, long-shelf-life snack in the market and is liked by low-income target groups [11]. However, few studies have compared the changes that occur in brown and refined rice (RR) after puffing.

In addition, volatile components are considered to be the most significant characteristics of PR. Volatile components are also related to varietal differences and degree of processing. In general, more aroma substances of the native rice (NR) and the finished products (puffed brown and refined rice) originated mainly from the Maillard reaction and lipid oxidation. Previous studies have shown that volatiles are predominantly distributed in the bran layer of rice, and that the content of volatiles, especially alcohols, is significantly reduced after milling [12]. Cui et al. [13] showed that because the bran layer of BR retains volatiles, milling into RR reduced volatile components. In particular, milling significantly reduced the content of acids, esters, and alcohols in BR. Additionally, puffing brings out creamy, nutty, and roasted flavors. These volatile components give PR a pleasant aroma, which greatly influences consumer choice and purchase [14]. Sui et al. [15] demonstrated that puffing increased aldehyde content, and aldehydes usually produce a desirable aroma with grassy and fatty notes. Recently, in order to detect complex volatile components in food, electronic nose, gas chromatography-mass spectrometry (GC-MS), and gas chromatography-ion migration spectrometry (GC-IMS) have been widely applied. [16]. Solid-phase microextraction (SPME) offers sensitivity, cost-effectiveness, and automation advantages, making headspace solid-phase micro-extraction gas chromatograph-mass spectrometry (HS-SPME-GC-MS) especially suitable for identifying volatile compounds [17]. Currently, the puffing process of rice is mainly focused on optimizing process conditions and researching puffed rice products. There are few studies on the effect of puffing of different varieties of BR and RR on the physical properties and volatile components of rice.

This study selected two rice varieties as research subjects, systematically investigating the basic nutritional composition, microstructure, and molecular properties of the native rice (BR and RR) and the finished products (puffed brown and refined rice). Meanwhile, this study tracked and monitored the key volatile components of native and puffing rice by using GC-MS.

## 2. Materials and Methods

### 2.1. Materials

Two rice varieties were provided from Shanghai Academy of Agricultural Sciences (Shanghai, China). The samples were designated as 450C (Huxiangruan 450 brown rice), 450CH (Huxiangruan 450 puffed brown rice), 450J (Huxiangruan 450 refined rice), 450JH (Huxiangruan 450 puffed refined rice), 1212C (Huruan 1212 brown rice), 1212CH (Huruan 1212 puffed brown rice), 1212J (Huruan 1212 refined rice), and 1212JH (Huruan 1212 puffed refined rice).

### 2.2. Preparation of Puffed Rice

Two rice varieties were put into the puffer (Five Star Brand, Luoyang Haoruida Machinery Factory, Luoyang, China), respectively, and heated evenly. When the pressure reached 8–10 Mpa, the lid was opened to obtain the BR and RR of Huruan 1212 and Huxiangruan 450 after puffing.

### 2.3. Physicochemical Analysis

Moisture was measured using the direct drying method in the Chinese national standard GB 5009.3-2016. Starch was determined by using a Starch Assay Kit (AKSU015C, Beijing Boxbio Science Technology Co., Ltd., Beijing, China); fat was calculated using the Soxhlet extraction method in the Chinese national standard GB 5009.6-2016; protein was determined using the combustion nitrogen method in the Chinese national standard GB 5009.5-2016.

### 2.4. Scanning Electron Microscopy Analysis

An appropriate rice sample was tiled on a sample stub with conductive adhesive, and the excess powder sample was blown away. Then, the surface of the sample was sprayed with a gold layer. The microstructure of the sample was observed by Scanning Electron Microscopy (SEM) (TM4000 Plus, Hitachi, Tokyo, Japan) at 500×, respectively.

### 2.5. X-Ray Diffraction Analysis

According to the methods of Yan [18] et al., slightly modified, the rice starch was measured by an X-ray diffractometer (TD-3500, Dandong Tongda Technology Co., Ltd., Dandong, Liaoning, China) operated under 40 mA Cu-Kα radiation. The diffraction angle (2θ) ranged from 0° to 60° at a speed of 2°/min and a step size of 0.02°.

### 2.6. Fourier Transform Infrared Spectrum Measurements

The short-range ordered structure of the sample was determined using a Fourier transform infrared spectrometer (912A1139, Thermo Fisher Scientific Co., Ltd., Shanghai, China) according to the method of Chen et al. [19]. The rice flour sample was pressed into thin slices. The IR spectra were scanned from 500 to 4000 cm^−1^ at a resolution of 4 cm^−1^ and recorded with 64 scans.

### 2.7. Thermogravimetry Analysis

The rice sample was ground into rice flour and passed through a 100 μm sieve, and the rice sample (about 10 mg) was placed in a crucible. The empty crucible was used as a control group in an air atmosphere, and the heating rate was set at 10 °C min^−1^ so that the thermogravimetric analyzer (DZ-TGA101, Nanjing Dazhan Testing Instrument Co., Ltd., Nanjing, China) was raised from room temperature to 600 °C.

### 2.8. Sensory Evaluation

For more accurate sensory qualities, eight members between the ages of 20–30 years were assembled. The evaluators had a thorough knowledge of the study prior to conducting the study. While tasting samples, each member was given drinking water to rinse his/her oral cavity between consumption of the samples. The total score was set at 100 points, and the scores were divided into six aspects: color (20 points), appearance (20 points), taste (20 points), flavor (20 points), shell hardness (10 points), and overall acceptability (10 points). The average score of 8 people on the total score of each index was used as the result. The sensory evaluation score is shown in Table A1.

### 2.9. GC-MS Analysis

The volatile components of rice before and after being puffed were slightly modified according to the method of Li et al. [20]. The bottle was sealed after 2 g of sample rice and 5 μL of 0.04 mg/mL 2-methyl-3-heptanone were added to a 15 mL brown extraction bottle. Solid-phase microextraction (SPME) fibers (DVB/CAR/PDMS, 50/30 μm, 1 cm) were exposed to headspace air at 80 °C for 30 min. The SPME fiber was inserted into the injection port of a gas chromatography-mass spectrometer (7200, Agilent Technologies Co., Ltd., Santa Clara, CA, USA) and desorbed at 250 °C for 5 min. The split mode (5:1) was used for injection. The DB-WAX column (30 m × 0.25 mm × 0.25 μm, Agilent Technologies Co., Ltd., Santa Clara, CA, USA) was used to separate and evaluate the volatile components with high-purity helium (purity > 99.999%) as the carrier gas at a flow rate of 1 mL/min. The column temperature was set at 40 °C for 5 min and then increased to 230 °C at 5 °C/min for 10 min. The mass selective detector was operated in electron bombardment ionization mode (70 eV) with a scan range of 40–500 *m*/*z*. The temperature of the ion source was 230 °C. All experiments were repeated three times. The NIST17 standard library was selected and the sample peaks were qualitatively analyzed by comparative mass spectrometry (MS) and retention index (RI), and the relative content of compounds was quantified based on the peak area.

The flavors in the samples were assessed according to the relative odor activity value (ROVA) of Wu et al. [21]. The one that contributes the most to the overall flavor of the sample was set to 100, and the others were calculated according to Equation (1).

(1) $${\mathrm{ROVA}}_{\left(i\right)}\;\approx \;\frac{100\times C_{i}\times T_{stan}}{C_{stan}\times T_{i}}$$

where *C_i_* denotes the relative content of each volatile component; *C_stan_* denotes the relative content of the compound that contributes the most to the overall aroma of the sample; *T_i_* is the sensory threshold corresponding to each volatile component; and *T_stan_* is the sensory threshold corresponding to the compound that contributes the most to the overall aroma of the sample.

### 2.10. Statistical Analysis

All experiments were repeated three times, and the data were expressed as mean ± standard deviation. SPSS 26.0 statistical software (SPSS Inc., Chicago, IL, USA) was used for significance analysis. Statistical significance was assessed using analysis of variance (ANOVA). Different English letters indicated significant difference (*p* < 0.05). Data analysis and figure drawing was performed using Origin 2024 software (Origin Lab Corporation, Northampton, MA, USA).

## 3. Results

### 3.1. Analysis of Physicochemical Components

The moisture content of all the PR ranged between 6.42% and 7.50%, while that of NR decreased from 12.6% and 14.63% to 7.05% and 7.23% due to high-temperature treatment during puffing (Table 1). The moisture content of PR significantly decreased due to the evaporation of moisture during the high-temperature puffing [22]. The low moisture content of PR prevents microbial contamination and extends food shelf life. More importantly, the moisture content of PR is extremely critical from a sensory point of view. This is because PR is a crispy product, and its moisture content greatly affects its crispiness. The two varieties of NR (450C, 450J, 1212C, and 1212J) and PR (450CH, 450JH, 1212CH, and 1212JH) starch and protein content were significantly different (*p* < 0.05), and puffing increased the starch and protein content on a wet basis but increased them little on a dry basis. The elevated starch content is attributed to structural disintegration of gelatinized starch under high temperatures and pressures, where soluble amylopectin fragments and leached amylose increase the enzymatic hydrolysis rate [23]. The protein content of all the samples was higher than NR after puffing, possibly due to water evaporation and loss of water-soluble components [24]. Proteins and starch formed aggregated structures, which can inhibit the hydrolysis of amylase and thereby reduce digestibility [24]. The fat content of PR decreased compared to that of unprocessed rice, which may be attributed to fat decomposition into fatty acids and monoglycerides or partial evaporation under high-temperature and -pressure conditions during puffing [25].

Additionally, the starch content of RR was notably higher than that of BR, while the protein and fat content was significantly lower. This phenomenon may be because the hulls were removed as the degree of milling increased, resulting in a significant loss of protein and lipid content, while starch is present in endosperm, and its content increases after grinding into RR [26].

### 3.2. Microstructure Analysis

**Figure 1:**
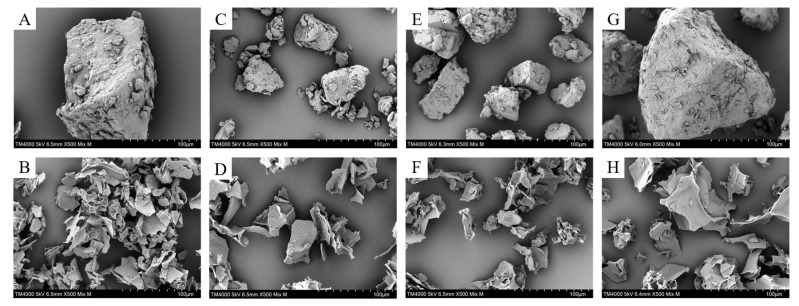
SEM images of different varieties of NR and PR at 500× magnification. The labels represent 450C (**A**), 450CH (**B**), 450J (**C**), 450JH (**D**), 1212C (**E**), 1212CH (**F**), 1212J (**E**), 1212JH (**H**), respectively.

As seen in Figure 1, NR maintained granule integrity and compact structure, while PR showed structural disruption, transitioning from a crystalline to an amorphous structure. High temperature and pressure were applied during the puffing process to degrade starch molecules, accelerate starch gelatinization, and form a damaged appearance [27]. Other researchers have reported similar observations that the high temperatures during puffing cause the water in the rice to flash evaporate and dry out rapidly, leading to a loss of flexibility in the structure and imparting a porous structure to the PR [11,28]. This evidence supports our experimental conclusion. Furthermore, studies have shown that starch undergoes degradation during the puffing process; the mechanisms underlying such transformations warrant systematic investigation in future research.

### 3.3. Relative Crystallinity Analysis

**Figure 2:**
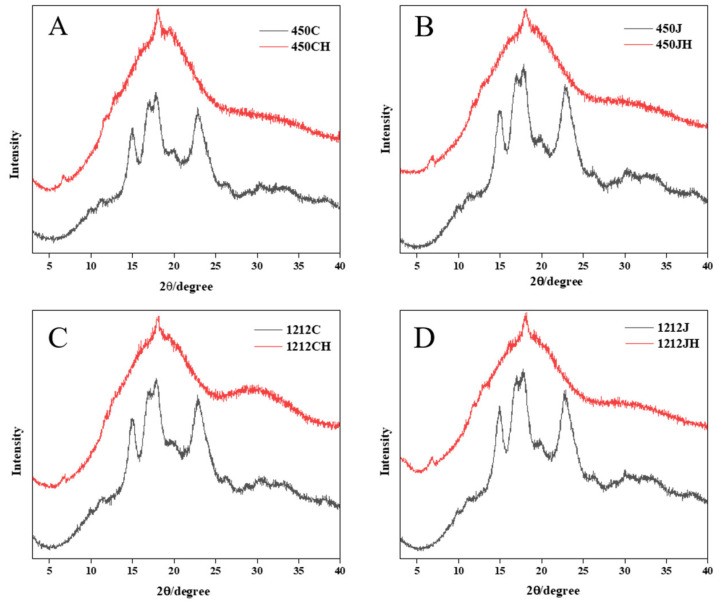
X-ray diffraction analysis of different varieties of NR and PR. (**A**): 450C and 450CH; (**B**): 450J and 450JH; (**C**): 1212C and 1212CH; (**D**): 1212J and 1212JH.

Starch crystal structures are commonly categorized into three types: A, B, and C, based on their unique diffraction peak. A-type starch exhibits prominent peaks at 15°, 17°, 18°, and 23°; B-type starch at 5.6°, 17°, 22°, and 24°; and C-type starch at 5.6°, 15°, 17°, 19°, 23°, and 26°. As seen in Figure 2, NR flour showed separate diffraction peaks at 15° and 23°, while continuous double diffraction peaks at 17° and 18°, indicating that the crystal structure of rice starch, are typical of a A-type crystal [29]. After puffing, rice starch displayed V-type diffraction peaks at 7°, 13°, and 20°, signifying a transition from an A-type to V-type crystal structure. This transformation is likely attributed to the formation of a starch–lipid complex between starch and fat [30]. This is also consistent with the results of Kuo [9] et al. when puffing rice flour. In addition, some studies have indicated that the alteration of PR crystallinity may be potentially correlated to the dextrinization process of starch, which involves the thermal breakdown of starch molecules into shorter dextrin chains. This transformation resulted in the formation of a more amorphous structure and reduced clarity of X-ray diffraction peaks [8]. Puffing not only induces starch gelatinization but also triggers dextrinization, further degrading the crystalline arrangement of starch. Compared to NR, PR diminished the peak intensity and increased amorphous configuration, suggesting a higher level of starch degradation and a more pronounced loss of structural crystallinity [8].

The crystallinity of 1212J and 1212C is higher than that of 450J and 450C (Table 2). Specifically, 1212JH exhibited the lowest crystallinity at 12.72% among the samples, whereas 1212C demonstrated a high level of crystallinity at 26.83%. This phenomenon occurs because the amylose content decreases the crystallinity of the cereal matrix, while the higher amylopectin content increases the crystallinity of the cereal matrix [6]. Previous studies have also shown that the crystalline region of rice consists mainly of amylopectin, influenced by the fine structure, degree of polymerization and molecular weight [31]. Meanwhile, the above figure clearly shows that the crystallinity of rice flour after puffing is significantly reduced, which is consistent with the analysis of SEM results.

### 3.4. FT-IR Spectra Analysis

**Figure 3:**
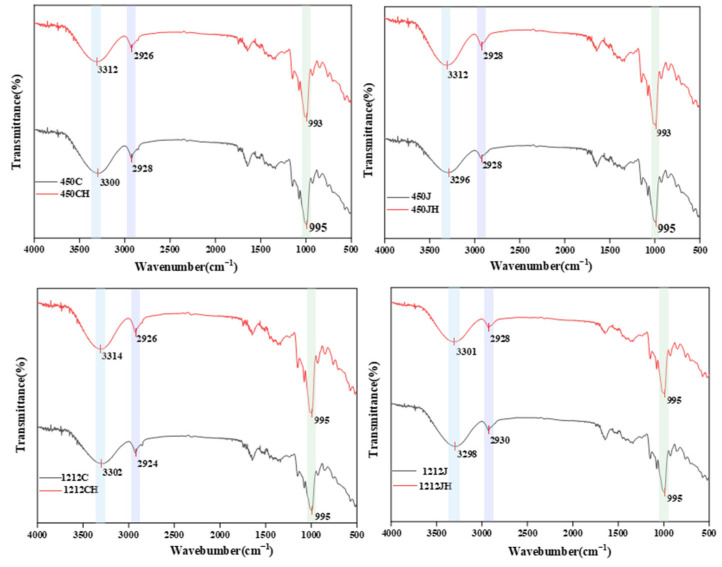
FT-IR analysis of different varieties of NR and PR. Blue for -OH stretching, purple for C-H stretching, green for C-O stretching.

FT-IR spectra represent helicity and crystallinity, reflecting the short-range order of molecules in the starch structure [32]. The FT-IR spectra of NR and PR exhibited distinct peaks across four wave number ranges: below 800 cm^−1^, 800–1500 cm^−1^, 1500–3000 cm^−1^, and 3000–3500 cm^−1^ (Figure 3). In particular, the spectrum of gelatinized starch is sensitive in the range of 900–1100 cm^−1^, a region that reflects the crystalline part of the starch in NR and PR, where the starch granules absorb energy during the pasting process, leading to disruption of the crystal structure and loss of the ordered structure [33]. A prominent peak at approximately 1000 cm^−1^ corresponds to the C-O stretching of starch glucose units [34], while peaks at 2800–3000 cm^−1^ are attributed to C-H stretching [35]. Similar peaks were observed for NR and PR, indicating that puffing treatment would only lead to physical modification and predicting no substantial bond changes in puffing conditions. Additionally, compared with non-treated BR, the peak of puffed brown rice at 3400 cm^−1^ was narrowed, indicating a potential reduction in the number of hydrogen bonds due to the expansion process, and the vibration stretching peak of -OH shifted slightly to a higher wavenumber. Analysis of IR spectra indicated that the puffing treatment did not alter chemical groups; however, puffing weakened hydrogen bond interactions and hindered recrystallization, thereby slowing the rate of retrogradation. This observation is consistent with the results of X-ray diffraction analysis.

### 3.5. Thermal Stability

**Figure 4:**
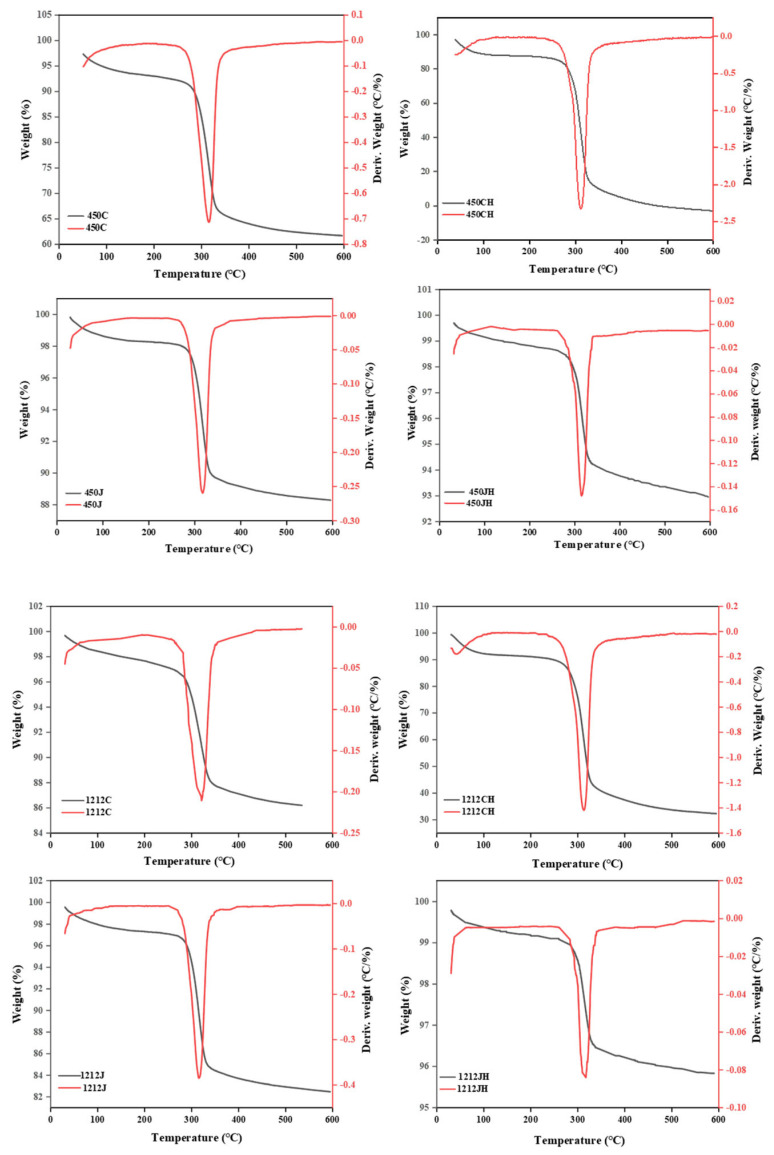
TGA of different varieties of NR and PR.

The thermal stability of the two varieties of NR and PR was assessed by thermogravimetric analysis (TGA), as shown in Figure 4. All samples showed a trend of mass loss. Different varieties of NR and PR exhibited two stages of weight loss. The first stage was 30–200 °C. The mass loss is related to the removal of moisture [6]. The second stage occurred at 200–600 °C, which may be attributed to the degradation of starch granules. Specifically, the maximum mass loss of all samples was observed between 250 and 300 °C. The results are consistent with previous literature observations [36]. Some of the starch granules had been degraded during the preparation of PR. Consequently, the decomposition peak of the PR transferred to a lower temperature. The residual starch content of the puffed refined rice after decomposition was greater than that of the RR, while puffed brown rice exhibited reduced residual starch compared to BR. This phenomenon is associated with the amylose content in starch granules, their crystallinity, and granule size [31], which is also consistent with the results of SEM analysis.

### 3.6. Sensory Quality of Native and Puffing Rice

**Figure 5:**
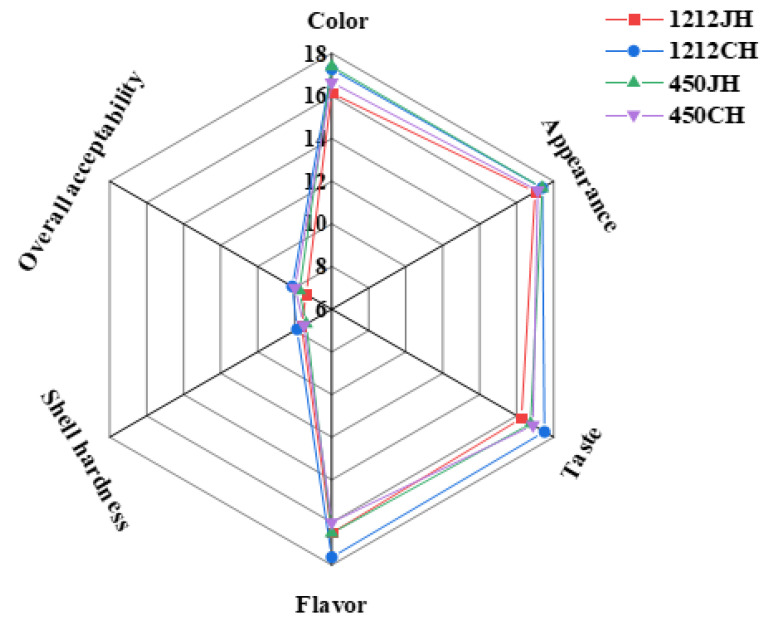
Sensory evaluation and texture properties of different varieties of NR and PR.

Puffing technology significantly affected the appearance, color, and taste of rice flour. NR and RR show significant differences (*p* < 0.05) in the different varieties of PR (Figure 5). Since BR contains a bran layer, the puffed brown rice color was caramel and scored low for color and appearance. However, the outer layer of BR was removed from the RR, which improved the overall appearance quality of the puffed refined rice. Among the samples evaluated, 1212CH received the highest overall acceptability score of 8.13 ± 0.99. In terms of taste, 1212CH also obtained the highest rating. The higher hardness of puffed brown rice than puffed refined rice may be because BR retains the bran layer. In contrast, RR has a higher starch content. Puffing led to starch gelatinization, thus destroying the structure of starch. Starch gelatinization forms a gelatinous substance that makes the structure of the rice fluffy, thus reducing hardness. In addition, puffing produces a number of volatile components, including aldehydes, ketones, alcohols, esters, and pyrazines, which intensify the flavor of the rice. Different varieties of rice produce unique aromas after puffing, and these compounds partially mask the flavor of the rice bran of BR, giving 1212CH a maximum flavor sensory score of 17.63 ± 1.85.

### 3.7. GC-MS Analysis of Volatile Compounds in Native and Puffing Rice

**Figure 6:**
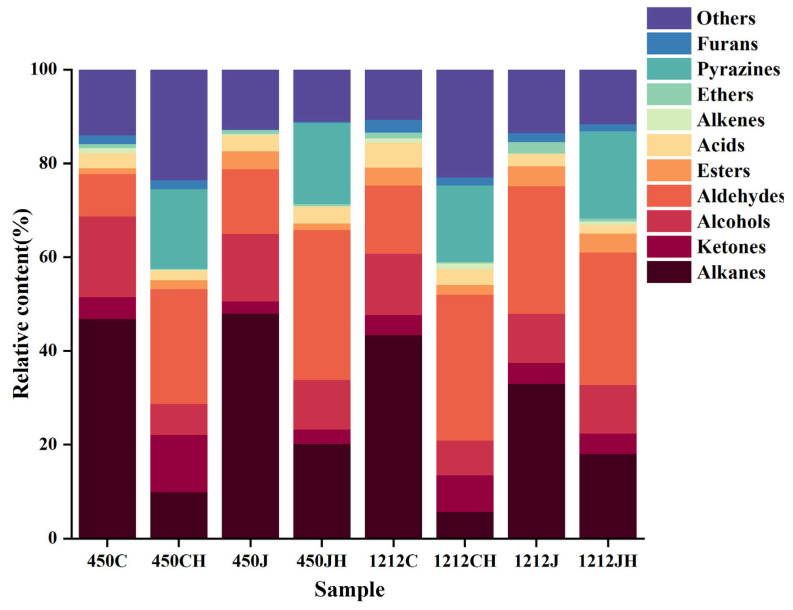
Volatile flavor components of different varieties of NR and PR.

The volatile components in different varieties of NR and PR were analyzed using the SPME-GC-MS technology (Figure 6). The volatile components include alkanes, ketones, alcohols, aldehydes, esters, acids, olefins, ethers, pyrazines, and furans, among others. The relative content of volatile components in PR was higher than in NR. One possible reason is that puffing increases pyrazines in the rice, which are derived from a Maillard reaction between amino acids and carbohydrates, and which provide the PR with a toasted and nutty flavor [37]. Additionally, the high-temperature-caused differences following the puffing process led to the cracking of alkanes, thereby reducing their content. Most alkanes have a high flavor threshold but do not have a significant effect on overall flavor. In contrast, the puffing technology increased the content of aldehydes and ketones. Since some volatile components do not have a significant effect on the aroma of rice, this paper focuses only on the key volatile components in rice.

The 136 important volatile components are displayed in Table A2. Ketones, aldehydes, and alcohols were the main volatile components in NR and PR, followed by alkanes, acids, esters, alkenes, ethers, pyrazines, furans, and others. However, 36, 60, 29, 51, 35, 58, 33, and 55 volatiles were detected in 450C, 450CH, 450J, 450JH, 1212C, 1212CH, 1212J, and 1212JH. This variation is attributed to differences in varieties, degree of processing, and methods of analyzing volatiles [38]. It can be seen that BR exhibits a pronounced aroma compared to RR, possibly as volatile substances are mainly present in the bran of BR, whereas the milling of BR into RR results in reduced levels of substances such as alcohols and ketones. [39]. Meanwhile, we calculated the ROVA for volatiles and summarized the top ten key volatile constituents (Table A3).

Aldehydes are recognized as the primary flavor constituents in cereals, primarily originating from the oxidation and decomposition of fatty acids [31]. Nonanal has been reported to originate mainly from the oxidative breakdown of unsaturated fatty acids such as oleic acid. Nonanal contributes flavors such as citrus, cucumber, floral, freshness, grassy, soapy, and fatty flavors [12]. The decrease in the nonanal content of puffed brown rice may be due to the fact that proteins, starch, and other matrices in BR may interact with volatile compounds to hinder flavor release [40], whereas the nonanal content of RR increased, which may be related to the degree of processing and the variety and source of the rice [41]. As seen in Table A2, the benzaldehyde content increased after puffing, which may be attributed to the combined effect of the Maillard reaction and lipid oxidation. Notably, benzaldehyde may contribute to the sweet aromatic character of PR [42].

Alcohols are considered secondary products of unsaturated fatty acid oxidation, which are formed by the further decomposition of aldehydes; degradation of carbohydrates also produces large amounts of alcohols [12]. BR has a higher relative content of alcohol compared to RR. 2-Furanmethanol and 1-octen-3-ol are the most abundant volatile alcohol and effective aromatic compound. 2-furanmethanol is detected mainly in PR, with moldy, sweet caramel bread and coffee flavors [43]. 1-Octen-3-ol has a strong soil and sweet and herbaceous odor, and the content of 1-octen-3-ol decreased after puffing. The probable reason is related to the high temperatures and pressures of puffing [12,31].

Ketones are products of unsaturated fatty acid oxidation and amino acid degradation. These compounds have a creamy fruity flavor, and some studies suggest that ketones exhibit a mild sweet and sour aroma [39]. 6-Methyl-5-hepten-2-one is the main volatile ketone body, providing fruity and floral aromas [44].

Esters are volatile components with a fruity aroma and they have a positive effect on the flavor and organoleptic properties of foods. All the samples contained low levels of esters, contributing a relatively low value to aroma [39].

In addition, 2-acetyl-1-pyrroline (2-AP) is considered one of the key flavors that provides rice with the aroma of popcorn and toasted bread produced during the growing process. However, Wang et al. [42] found that 2-AP was not detected in samples after high-temperature treatment, which suggests that 2-AP may be derived from the rice itself rather than from components produced by the Maillard reaction. Some studies have found that 2-acetyl-1-pyrroline is not significantly produced during the cooking process, which is also consistent with our study [39]. In general, BR contains higher levels of 2-acetyl-1-pyrroline than RR, probably because 2-acetyl-1-pyrroline can be linked to lipid compounds, and BR has a higher fat content than RR.

There are also unique aroma components between different groups. Among them, the unique aroma components in 450CH were furaneol (0.9%), 2,5-dimethylfuran-3,4(2H,5H)-dione (0.9%), and 4-ethoxy-3-anisaldehyde (1.43%). These volatile components are usually characterized by a caramel and sweet taste. Several unique volatile components, such as 2-thiophenecarboxaldehyde (0.5%) and 2(1H)-pyridinone (0.54%), were found in 450JH. These volatile components contribute primarily to complex aromas such as roasted flavors and coffee. The characteristic volatile components, (E)-2-octenal, (0.5%), acetic acid (1.82%), 4-vinylphenol (1.09%), and 2-methoxy-phenol (0.98%), were identified in 1212CH. Among them, (E)-2-octenal is produced by the oxidation and decomposition of oleic acid providing fruity, floral, and sweet flavors. 4-Vinylphenol was recognized as a product of the thermal degradation of phenolic acids. 4-Vinylphenol was characterized by an unpleasant smell of rotting straw odor. In 1212CH sample, 4-vinylphenol contained significantly high concentrations. This is also associated with bran in BR [42]. In addition, the characteristic volatile components in 1212JH were cyclododecanol (0.48%) and nerolidol, cis- (0.51%). Overall, these characteristic flavor substances contribute to the aromas of NR and PR and distinguish between different BR and RR varieties.

Similarly, untreated BR and RR also have unique flavor components compared to PR. 2,3-dimethyl-cyclohexanol (0.61%), and 2,3-butanediol (1.72%) were detected in 450C. These volatiles mainly provided a creamy sweet flavor. N-Caproic acid vinyl ester (0.20%) and 9,12-octadecadienoic acid (Z, Z)- (0.22%) were identified from 450J. N-Caproic acid vinyl ester has a sweet aroma similar to banana and pineapple. Trans-2-Undecen-1-ol (0.43%) and oleic acid (0.34%) were found in 1212C. Trans-2-Undecen-1-ol had a grassy and fruity flavor, (Z)-2-heptenal offered a strong grassy and cucumber-peel aroma. The compounds 2-nonanone (0.31%) and 2-(octadecyloxy)-ethanol (0.32%) were found in 1212J. At low concentrations, 2-nonanone exhibited fruity aromas of melon and banana.

In summary, key aroma substances were identified by volatile odor and aromatic compounds These compounds were related to the variety, the degree of processing, and the puffing technology.

## 4. Conclusions

In summary, this study evaluated varietal differences in physicochemical properties and volatile organic compounds between BR and RR, with further analysis of how puffing technology affects these properties. The findings revealed significant alterations, with puffing causing reduced fat and moisture content in BR and RR. SEM analysis demonstrated that puffing disrupted the structural integrity of starch, causing starch granules to transition from a regular configuration to a disordered, loose morphology. According to XRD and FT-IR spectroscopy, the swelling process produced starch–lipid complexes forming a V-shaped structure, reducing the crystallinity of starch and inhibiting starch recrystallization but not generating new chemical groups. In addition, 136 key volatile components were detected by GC-MS, with aldehydes and ketones considered to be the main volatile components of PR. In particular, puffing produced pyrazines, which increased the roasted and nutty flavors in the rice but reduced the alcohols containing floral and fruity flavors and the esters containing fruit aromas. The bran of BR is rich in proteins and fats; puffed brown rice contains more volatile components, most notably its ketone content. Overall, the aroma of rice was made stronger and more prosperous by puffing brown and refined rice. By determining and characterizing the physicochemical properties and key volatile components of NR and PR, the findings can be used as a reference for PR products. Since only one technology was used to alter rice, future research could combine puffing technology with other technologies such as fermentation, microwave, and enzymatic hydrolysis to further improve the quality of PR products.

## Figures and Tables

**Table 1:** Physicochemical components in NR and PR.

Varieties	Moisture	Starch	Protein	Fat
1212J	14.63 ± 0.29 ^c^	59.34 ± 0.26 ^d^	6.28 ± 0.14 ^a^	0.54 ± 0.10 ^a^
1212JH	7.50 ± 0.04 ^a^	61.53 ± 1.54 ^e^	7.72 ± 0.03 ^d^	0.11 ± 0.00 ^a^
1212C	13.10 ± 1.36 ^b^	51.80 ± 0.13 ^a^	7.4 ± 0.2 ^c^	2.55 ± 0.52 ^c^
1212CH	7.23 ± 0.02 ^a^	57.02 ± 0.73 ^bc^	8.63 ± 0.03 ^f^	1.98 ± 0.03 ^b^
450J	12.82 ± 0.02 ^b^	61.47 ± 0.37 ^e^	6.89 ± 0.15 ^b^	0.40 ± 0.04 ^a^
450JH	6.42 ± 0.09 ^a^	62.25 ± 0.25 ^e^	7.70 ± 0.05 ^cd^	0.35 ± 0.00 ^a^
450C	12.60 ± 0.15 ^b^	55.57 ± 0.06 ^b^	8.12 ± 0.04 ^e^	2.23 ± 0.05 ^bc^
450CH	7.05 ± 0.08 ^a^	58.01 ± 1.32 ^cd^	8.99 ± 0.03 ^g^	1.97 ± 0.02 ^b^

Note: Different lower-case letters in the same column indicate significant differences (*p* < 0.05). The following table is the same.

**Table 2:** Crystallinity of NR and PR.

Varieties	Relative Crystallinity (%)
1212J	24.20 ± 1.45
1212JH	12.72 ± 1.39
1212C	26.83 ± 2.93
1212CH	15.97 ± 0.19
450J	20.90 ± 0.90
450JH	14.17 ± 1.02
450C	22.11 ± 3.22
450CH	14.78 ± 1.08

## Data Availability

The original contributions presented in the study are included in the article, further inquiries can be directed to the corresponding author.

## Appendix A

**Table A1:** Sensory evaluation table of rice before and after puffing.

Program	Total	Standard	
		Refined Rice	Brown Rice
Color	20	White and uniform (17-20) Yellowish and uniform (13-16) Burnt yellow and uniform (10-12)	Caramel color, texture rules (17-20) Deepened caramel color and uniform (13-16) Dark brown, uneven color (10-12)
Appearance	20	Full shape and consistent the overall appearance (17-20) Part not all brown open (13-16) A large number of not all blow up (10-12)	Full shape and consistent the overall appearance (17-20) Part not all brown open (13-16) A large number of not all blow up (10-12)
Taste	20	Consistent the inside and outside, tender and crisp (17-20) Uniform the taste (13-16) Internal cotton flocculent, uneven taste (10-12)	Consistent the inside and outside, tender and crisp (17-20) Uniform the taste (13-16) Internal cotton flocculent, uneven taste (10-12)
Flavor	20	Full the taste and thick the aftertaste (17-20) More aftertaste (13-16) Flat the taste and no aftertaste (10-13)	Full the taste and thick the aftertaste (17-20) More aftertaste (13-16) Flat the taste and no aftertaste (10-13)
Shell hardness	10	High brittleness for 10 score The brittleness difference for 0 score	High brittleness for 10 score The brittleness difference for 0 score
Overall acceptability	10	Highly acceptable for 10 score Low acceptability for 0 score	Highly acceptable for 10 score Low acceptability for 0 score

**Table A2:** The flavor substances and relative contents of raw rice and puffed rice.

No	CAS	Volatile Compounds	Formula	Relative Amount (%)							
				450C	450CH	450J	450JH	1212C	1212CH	1212J	1212JH
Alkanes											
1	112-40-3	Dodecane	C12H26	2.59 ± 0.34	1.29 ± 0.43	1.29 ± 1.20	1.44 ± 0.56	7.25 ± 0.49	0.72 ± 0.07	1.83 ± 0.24	0.41 ± 0.25
2	556-67-2	Cyclotetrasiloxane, octamethyl-	C8H24O4Si4	1.61 ± 0.87	0.36 ± 0.05	1.92 ± 0.23	0.99 ± 0.53	1.50 ± 0.07	0.26 ± 0.03	1.29 ± 0.16	0.86 ± 0.06
3	2847-72-5	Decane, 4-methyl-	C11H24	2.42 ± 0.05	0.25 ± 0.02	0.59 ± 0.18	0.21 ± 0.13	1.70 ± 0.71	0.07 ± 0.01	0.24 ± 0.11	0.10 ± 0.07
4	6117-97-1	Dodecane, 4-methyl-	C13H28	0.73 ± 0.13	0.40 ± 0.10	1.56 ± 0.33	0.59 ± 0.19	0.92 ± 0.18	0.15 ± 0.03	1.03 ± 0.28	0.30 ± 0.11
Ketones											
5	67-64-1	Acetone	C3H6O	1.01 ± 0.08	0.29 ± 0.01	0.65 ± 0.07	0.56 ± 0.16	0.71 ± 0.06	0.32 ± 0.10	0.58 ± 0.15	1.04 ± 0.02
6	116-09-6	1-Hydroxy-2-propanone,	C3H6O2	/	1.26 ± 0.03	/	1.31 ± 0.09	/	1.73 ± 0.05	/	1.48 ± 0.05
7	513-86-0	Acetoin	C4H8O2	/	0.20 ± 0.18	/	/	/	/	/	/
8	431-03-8	2,3-Butanedione	C4H6O2	/	0.14 ± 0.00	/	/	/	/	/	/
9	592-20-1	2-Propanone, 1-(acetyloxy)-	C5H8O3	/	0.30 ± 0.01	/	/	/	0.23 ± 0.01	/	/
10	930-60-9	4-Cyclopentene-1,3-dione	C5H4O2	/	0.02 ± 0.03	/	/	/	0.10 ± 0.01	/	/
11	1604-28-0	6-Methyl-3,5-heptadiene-2-one	C8H12O	0.17 ± 0.02	0.11 ± 0.02	/	/	0.14 ± 0.12	0.13 ± 0.02	/	/
12	22047-26-3	1-(6-Methyl-2-pyrazinyl)-1-ethanone	C7H8N2O	/	1.49 ± 0.30	/	/	/	1.49 ± 0.49	/	/
13	1004-37-1	2,6-Dimethyl-4-thiopyrone	C7H8OS	/	0.43 ± 0.00	/	/	/	/	/	/
14	689-67-8	5,9-Undecadien-2-one, 6,10-dimethyl-	C13H22O	0.58 ± 0.20	0.21 ± 0.19	0.45 ± 0.16	0.45 ± 0.05	0.71 ± 0.02	0.19 ± 0.02	1.14 ± 0.12	0.56 ± 0.49
15	3658-77-3	Furaneol	C6H8O3	/	0.90 ± 0.03	/	/	/	/	/	/
16	68755-49-7	2,5-Dimethylfuran-3,4(2H,5H)-dione	C6H8O3	/	0.90 ± 0.03	/	/	/	/	/	/
17	111-13-7	2-Octanone	C8H16O	0.13 ± 0.12	/	/	/	/	/	/	/
18	28564-83-2	2,3-Dihydro-3,5-dihydroxy-6-me	C6H8O4	/	4.26 ± 0.14	/	0.03 ± 0.03	/	1.75 ± 0.09	/	0.05 ± 0.04
19	18402-82-9	3-Octen-2-one, (E)-	C8H14O	0.23 ± 0.04	/	/	/	/	/	/	/
20	85-90-5	Methylchromone	C10H8O2	/	0.13 ± 0.11	/	/	/	/	/	/
21	110-93-0	6-Methyl-5-hepten-2-one	C8H14O	1.44 ± 0.07	/	0.15 ± 0.14	/	1.56 ± 0.04	/	1.23 ± 0.11	/
22	30086-02-3	3,5-Octadien-2-one, (E, E)-	C8H12O	0.12 ± 0.10	/	/	/	/	/	/	/
23	13297-35-3	3-Ethyl-1,2-dihydro-2-oxoquinoxaline	C10H10N2O	/	/	/	/	/	0.18 ± 0.15	/	/
24	616-45-5	2-Pyrrolidinone	C4H7NO	/	/	/	/	/	0.26 ± 0.01	/	0.09 ± 0.01
25	14309-57-0	3-Nonen-2-one	C9H16O	/	/	/	/	/	0.21 ± 0.01	/	/
26	1125-21-9	2,6,6-Trimethyl-2-cyclohexene-1,4-dione	C9H12O2	/	/	/	/	/	0.13 ± 0.00	/	/
27	821-55-6	2-Nonanone	C9H18O	/	/	/	/	/	/	0.31 ± 0.01	/
Alcohols											
28	14852-31-4	2-Hexadecanol	C16H34O	0.52 ± 0.22	0.25 ± 0.07	0.44 ± 0.09	0.11 ± 0.08	0.40 ± 0.32	0.24 ± 0.01	0.14 ± 0.03	0.24 ± 0.13
29	3391-86-4	1-Octen-3-ol	C8H16O	1.47 ± 0.17	0.13 ± 0.01	1.74 ± 0.26	0.40 ± 0.34	1.47 ± 0.32	0.15 ± 0.01	2.57 ± 0.19	0.52 ± 0.46
30	100-55-0	Nicotinyl alcohol	C6H7NO	/	0.34 ± 0.00	/	/	/	/	/	/
31	67-56-1	Methyl Alcohol	CH4O	/	0.31 ± 0.02	/	0.24 ± 0.06	/	0.23 ± 0.05	/	0.20 ± 0.17
32	64-17-5	Ethanol	C2H6O	1.79 ± 0.12	0.36 ± 0.02	1.42 ± 0.35	0.92 ± 0.20	1.39 ± 0.05	0.33 ± 0.07	1.53 ± 0.26	1.02 ± 0.04
33	2490-48-4	1-Hexadecanol, 2-methyl-	C17H36O	0.47 ± 0.08	0.20 ± 0.10	0.67 ± 0.18	0.35 ± 0.16	0.83 ± 0.04	0.16 ± 0.04	0.89 ± 0.31	0.20 ± 0.04
34	3857-25-8	2-Furanmethanol, 5-methyl-	C6H8O2	/	0.25 ± 0.01	/	/	/	0.19 ± 0.00	/	/
35	10042-59-8	2-Propyl-1-Heptanol	C10H22O	0.40 ± 0.34	/	/	/	/	/	/	/
36	98-00-0	2-Furanmethanol	C5H6O2	/	2.61 ± 2.26	/	6.42 ± 0.65	/	3.30 ± 2.86	/	3.69 ± 3.20
37	1724-39-6	Cyclododecanol	C12H24O	/	/	/	/	/	/	/	0.48 ± 0.42
38	1502-24-5	Cyclohexanol, 2,3-dimethyl-	C8H16O	0.61 ± 0.23	/	/	/	/	/	/	/
39	513-85-9	2,3-Butanediol	C4H10O2	1.72 ± 0.96	/	/	/	/	/	/	/
40	111-87-5	1-Octanol	C8H18O	3.60 ± 0.18	/	/	/	3.08 ± 0.07	/	/	/
41	5715-23-1	3,4-Dimethylcyclohexanol	C8H16O	0.24 ± 0.21	/	/	/	/	/	/	/
42	143-08-8	1-Nonanol	C9H20O	0.83 ± 0.04	/	1.24 ± 0.35	±	0.70 ± 0.04	/	0.63 ± 0.01	/
43	58670-89-6	2-Decyl-1-tetradecanol	C24H50O	/	/	0.89 ± 0.24	/	/	/	0.45 ± 0.39	/
44	112-53-8	1-Dodecanol	C12H26O	/	/	/	0.09 ± 0.08	/	/	/	0.15 ± 0.13
45	110-98-5	2-Propanol, 1,1’-oxybis-	C6H14O3	/	/	/	0.16 ± 0.02	/	/	/	/
46	636-72-6	2-Thiophenemethanol	C5H6OS	/	/	/	0.36 ± 0.05	/	/	/	/
47	515-03-7	1-Naphthalenepropanol, à-ethenyldecahydro-2-hydroxy-à,2,5,5,8a-pentamethyl-, [1R-[1à(R*),2á,4aá,8aà]]-	C20H36O2	/	/	0.17 ± 0.17	/	/	/	/	/
48	88728-58-9	(1aR,4S,4aR,7R,7aS,7bS)-1,1,4,7-Tetramethyldecahydro-1H-cyclopropa[e]azulen-4-ol	C15H26O	/	/	/	/	0.11 ± 0.10	/	/	/
49	75039-84-8	trans-2-Undecen-1-ol	C11H22O	/	/	/	/	0.43 ± 0.38	/	/	/
50	23238-40-6	2-(Decyloxy)ethanol	C12H26O2	/	/	/	/	/	/	0.18 ± 0.02	/
51	2136-72-3	2-(Octadecyloxy)-ethanol	C20H42O2	/	/	/	/	/	/	0.32 ± 0.14	/
52	142-50-7	Nerolidol, cis-	C15H26O	/	/	/	/	/	/	/	0.51 ± 0.02
53	629-96-9	1-Eicosanol	C20H42O	/	/	/	/	/	/	/	0.37 ± 0.07
Aldehydes											
54	620-02-0	2-Furancarboxaldehyde, 5-methyl-	C6H6O2	/	4.27 ± 0.05	/	0.69 ± 0.04	/	3.66 ± 0.02	/	0.33 ± 0.29
55	100-52-7	Benzaldehyde	C7H6O	0.15 ± 0.02	1.63 ± 0.02	0.24 ± 0.02	1.50 ± 0.02	0.11 ± 0.09	2.68 ± 0.17	0.60 ± 0.03	3.55 ± 0.06
56	112-31-2	Decanal	C10H20O	/	0.31 ± 0.02	/	1.95 ± 0.39	/	0.66 ± 0.57	/	2.75 ± 0.25
57	124-19-6	Nonanal	C9H18O	6.28 ± 0.18	2.88 ± 1.03	7.86 ± 2.17	14.63 ± 0.71	11.95 ± 0.17	3.47 ± 0.16	15.05 ± 1.24	13.29 ± 1.53
58	98-01-1	Furfural	C5H4O2	/	10.13 ± 0.19	/	3.34 ± 0.14	/	11.16 ± 0.29	/	2.22 ± 0.09
59	1003-29-8	1H-Pyrrole-2-carboxaldehyde	C5H5NO	/	1.13 ± 0.02	/	1.09 ± 0.04	/	2.48 ± 0.20	/	0.45 ± 0.03
60	120-25-2	4-Ethoxy-3-anisaldehyde	C10H12O3	/	1.43 ± 0.09	/	/	/	/	/	/
61	56599-95-2	Octadecanal, 2-bromo-	C18H35BrO	/	/	0.21 ± 0.08	0.07 ± 0.01	/	/	0.05 ± 0.04	0.06 ± 0.05
62	2548-87-0	2-Octenal, (E)-	C8H14O	/	/	/	/	/	0.50 ± 0.04	/	/
63	98-03-3	2-Thiophenecarboxaldehyde	C5H4OS	/	/	/	0.50 ± 0.03	/	/	/	/
64	57266-86-1	(Z)-2-Heptenal	C7H12O	/	/	/	/	0.19 ± 0.02	/	/	/
65	498-62-4	3-Thiophenecarboxaldehyde	C5H4OS	/	/	/	/	/	/	/	0.37 ± 0.32
Esters											
66	18202-24-9	10,13-Octadecadiynoic acid, methyl ester	C19H30O2	/	0.10 ± 0.08	/	/	/	0.06 ± 0.08	/	/
67	57156-91-9	2,5-Octadecadiynoic acid, methyl ester	C19H30O2	/	0.16 ± 0.02	/	/	/	/	/	/
68	56687-68-4	[1,1’-Bicyclopropyl]-2-octanoic acid, 2’-hexyl-, methyl ester	C21H38O2	0.14 ± 0.04	0.18 ± 0.14	0.11 ± 0.12	0.21 ± 0.19	0.12 ± 0.06	0.13 ± 0.01	0.17 ± 0.05	0.41 ± 0.11
69	112-23-2	Formic acid, heptyl ester	C8H16O2	0.20 ± 0.18	/	1.43 ± 0.88	/	/	/	/	/
70	112-32-3	Formic acid, octyl ester	C9H18O2	/	/	/	/	/	/	1.15 ± 0.09	1.21 ± 0.07
71	2046-21-1	Methyl 6-oxoheptanoate	C8H14O3	/	0.13 ± 0.11	/	/	/	/	/	/
72	13058-52-1	Methyl 9-cis,11-trans-octadecadienoate	C19H34O2	/	/	/	/	/	/	/	/
73	544-35-4	Linoleic acid ethyl ester	C20H36O2	0.11 ± 0.09	/	0.02 ± 0.01	/	0.16 ± 0.01	/	0.04 ± 0.01	/
74	3050-69-9	n-Caproic acid vinyl ester	C8H14O2	/	/	0.20 ± 0.18	/	/	/	/	/
75	7493-69-8	Allyl 2-ethyl butyrate	C9H16O2	/	/	/	/	0.16 ± 0.14	/	/	/
76	2305-05-7	ç-Dodecalactone	C12H22O2	/	/	/	/	/	0.15 ± 0.01	/	/
77	142-91-6	Isopropyl palmitate	C19H38O2	/	/	/	/	/	/	0.19 ± 0.00	0.26 ± 0.03
78	2306-78-7	Nerolidyl acetate	C17H28O2	/	/	/	/	/	/	/	0.26 ± 0.22
Acids											
79	16714-85-5	10-Heptadecen-8-ynoic acid, methyl ester, (E)-	C18H30O2	/	0.10 ± 0.01	/	0.09 ± 0.02	/	/	/	/
80	142-62-1	Hexanoic acid	C6H12O2	/	0.48 ± 0.12	/	0.99 ± 0.05	/	0.51 ± 0.05	/	/
81	112-05-0	Nonanoic acid	C9H18O2	0.47 ± 0.10	0.43 ± 0.11	0.60 ± 0.58	1.16 ± 0.20	0.63 ± 0.10	0.37 ± 0.02	0.40 ± 0.35	0.26 ± 0.22
82	544-63-8	Tetradecanoic acid	C14H28O2	0.89 ± 0.18	0.14 ± 0.05	0.57 ± 0.44	0.06 ± 0.07	0.90 ± 0.25	0.20 ± 0.11	0.61 ± 0.20	0.52 ± 0.16
83	10024-70-1	Butanoic acid, 3-methoxy-	C5H10O3	/	/	/	0.10 ± 0.09	/	/	/	/
84	506-17-2	cis-Vaccenic acid	C18H34O2	/	/	/	0.17 ± 0.15	/	/	/	/
85	60-33-3	9,12-Octadecadienoic acid (Z,Z)-	C18H32O2	/	/	0.22 ± 0.19	/	/	/	/	/
86	112-80-1	Oleic Acid	C18H34O2	/	/	/	/	0.34 ± 0.30	/	/	/
87	64-19-7	Acetic acid	C2H4O2	/	/	/	/	/	1.82 ± 0.12	/	/
88	111-14-8	Heptanoic acid	C7H14O2	/	/	/	/	/	/	/	0.11 ± 0.01
Alkenes											
89	1120-36-1	1-Tetradecene	C14H28	0.26 ± 0.23	/	/	/	/	/	/	/
90	4984-01-4	1-Octene, 3,7-dimethyl-	C10H20	/	/	/	/	0.18 ± 0.16	/	/	/
91	2628-17-3	4-Vinylphenol	C8H8O	/	/	/	/	/	1.09 ± 0.94	/	/
92	87-44-5	Caryophyllene	C15H24	/	/	/	/	/	/	/	0.23 ± 0.20
Ethers											
93	3055-98-9	Octaethylene glycol monododecyl ether	C28H58O9	0.43 ± 0.15	0.19 ± 0.04	0.70 ± 0.33	0.09 ± 0.01	0.42 ± 0.09	0.23 ± 0.06	0.40 ± 0.12	0.27 ± 0.11
94	108-61-2	2,2’-Oxydipropanol	C6H14O3	/	/	/	0.30 ± 0.12	/	/	/	/
95	140-67-0	Estragole	C10H12O	/	/	/	/	0.60 ± 0.00	/	1.57 ± 0.08	/
96	3055-93-4	Diethylene glycol monododecyl ether	C16H34O3	/	/	/	/	/	/	0.13 ± 0.08	/
97	2179-59-1	Allyl propyl disulfide	C6H12S2	/	/	/	/	/	0.10 ± 0.08	/	/
98	10020-43-6	Ethanol, 2-(octyloxy)-	C10H22O2	/	/	/	/	/	/	0.18 ± 0.16	0.08 ± 0.07
99	107-98-2	2-Propanol, 1-methoxy-	C4H10O2	/	/	/	/	/	/	/	0.11 ± 0.01
Pyrazines											
100	64608-60-2	4-Methylpyrrolo[1,2-a] pyrazine	C8H8N2	/	0.21 ± 0.02	/	/	/	/	/	/
101	13925-03-6	2-Ethyl-6-methyl- Pyrazine	C7H10N2	/	1.54 ± 0.07	/	1.18 ± 1.02	/	/	/	1.30 ± 0.05
102	290-37-9	Pyrazine	C4H4N2	/	0.21 ± 0.19	/	0.24 ± 0.21	/	0.26 ± 0.03	/	1.06 ± 0.05
103	109-08-0	Methyl-pyrazine	C5H6N2	/	3.10 ± 0.08	/	5.90 ± 0.52	/	3.61 ± 0.23	/	6.08 ± 0.05
104	123-32-0	2,5-Dimethyl-pyrazine	C6H8N2	/	3.15 ± 0.04	/	4.37 ± 0.92	/	3.58 ± 0.28	/	4.58 ± 0.11
105	108-50-9	2,6-Dimethyl-pyrazine	C6H8N2	/	60.8	/	1.24 ± 1.10	/	2.73 ± 0.28	/	2.02 ± 0.04
106	13925-00-3	Ethyl-pyrazine	C6H8N2	/	0.79 ± 0.05	/	1.16 ± 0.11	/	0.71 ± 0.31	/	0.93 ± 0.03
107	5910-89-4	2,3-Dimethyl-pyrazine	C6H8N2	/	0.38 ± 0.02	/	0.39 ± 0.03	/	0.34 ± 0.02	/	0.52 ± 0.02
108	14667-55-1	Trimethyl-pyrazine	C7H10N2	/	1.14 ± 0.99	/	0.77 ± 0.67	/	1.43 ± 0.11	/	0.95 ± 0.82
109	13360-65-1	3-Ethyl-2,5-dimethyl-pyrazine	C8H12N2	/	3.19 ± 0.47	/	1.00 ± 0.23	/	2.01 ± 0.09	/	1.12 ± 0.07
110	18217-82-8	2-Methyl-5-(1-propenyl)-, (E)-pyrazine,	C8H10N2	/	0.22 ± 0.01	/	/	/	0.14 ± 0.01	/	/
111	13360-64-0	2-Ethyl-5-methyl-pyrazine,	C7H10N2	/	/	/	/	/	0.36 ± 0.32	/	/
Others											
112	97-53-0	Eugenol	C10H12O2	/	/	/	/	/	0.14 ± 0.01	/	/
113	90-05-1	2-Methoxy-phenol	C7H8O2	/	/	/	/	/	0.9813.24 ± 0.03	/	/
114	96-76-4	2,4-Di-tert-butylphenol	C14H22O	0.04 ± 0.02	/	0.10 ± 0.03	0.15 ± 0.02	0.15 ± 0.02	/	1.04 ± 0.10	0.88 ± 0.05
115	103-90-2	Acetaminophen	C8H9NO2	/	0.39 ± 0.23	/	/	/	/	/	/
116	128-37-0	Butylated Hydroxytoluene	C15H24O	/	/	/	0.23 ± 0.03	/	/	/	0.16 ± 0.01
117	1020-31-1	1,2-Benzenediol, 3,5-bis(1,1-dimethylethyl)-	C14H22O2	/	/	/	0.04 ± 0.03	/	/	/	0.08 ± 0.07
118	142-08-5	2(1H)-Pyridinone	C5H5NO	/	/	/	0.54 ± 0.47	/	/	/	/
119	2294-76-0	Pyridine, 2-pentyl-	C10H15N	/	/	/	/	/	0.25 ± 0.02	/	/
120	1122-62-9	Ethanone, 1-(2-pyridinyl)-	C7H7NO	/	0.14 ± 0.00	/	/	/	0.15 ± 0.02	/	/
121	1438-94-4	1H-Pyrrole, 1-(2-furanylmethyl)-	C9H9NO	/	0.88 ± 0.02	/	0.24 ± 0.01	/	0.88 ± 0.01	/	0.06 ± 0.05
122	1072-83-9	Ethanone, 1-(1H-pyrrol-2-yl)-	C6H7NO	0.07 ± 0.00	0.81 ± 0.02	0.06 ± 0.01	0.11 ± 0.00	/	0.96 ± 0.02	/	0.08 ± 0.01
123	19549-87-2	2,4-Dimethyl-1-heptene	C9H18	0.23 ± 0.03	/	/	/	0.06 ± 0.01	/	/	/
124	137-00-8	5-Thiazoleethanol, 4-methyl-	C6H9NOS	/	0.16 ± 0.02	/	/	/	0.10 ± 0.01	/	/
125	85213-22-5	2-Acetyl-1-pyrroline	C6H9NO	2.96 ± 0.13	/	1.47 ± 0.17	/	/	/	/	/
126	7467-91-6	6-Quinoxalinol	C8H6N2O	/	0.14 ± 0.12	/	/	/	/	/	/
127	95-16-9	Benzothiazole	C7H5NS	/	/	/	/	0.15 ± 0.00	/	/	/
128	541-58-2	2,4-Dimethyl-thiazole	C5H7NS	/	/	/	/	/	/	/	0.34 ± 0.10
129	106-46-7	1,4-Dichloro-Benzene	C6H4Cl2	/	/	/	/	0.61 ± 0.00	/	/	/
130	108-88-3	Toluene	C7H8	/	/	/	/	0.14 ± 0.12	/	0.09 ± 0.02	/
131	122-39-4	Diphenylamine	C12H11N	/	/	/	/	0.03 ± 0.00	/	0.24 ± 0.03	/
132	88-15-3	Ethanone, 1-(2-thienyl)-	C6H6OS	/	/	/	0.13 ± 0.11	/	/	/	0.12 ± 0.10
133	126-52-3	Ethinamate	C9H13NO2	0.38 ± 0.01	/	/	/	/	/	/	/
134	120-72-9	Indole	C8H7N	0.40 ± 0.02	0.20 ± 0.02	0.58 ± 0.07	0.12 ± 0.01	0.13 ± 0.01	0.13 ± 0.02	0.48 ± 0.01	0.13 ± 0.00

Note: / represent undetected.

**Table A3:** ROVA of the 10 key volatile compounds in all samples.

No	CAS	Compounds	Threshold (mg/kg)	ROVA							
				450C	450CH	450J	450JH	1212C	1212CH	1212J	1212JH
1	30086-02-3	3,5-Octadien-2-one, (E, E)-	0.15	89.61	/	/	/	/	/	/	/
2	64-17-5	Ethanol	7.8	25.71	0.00	2.32	0.02	1.49	0.09	1.30	0.98
3	100-52-7	Benzaldehyde	0.44	38.19	0.09	6.94	0.44	2.09	13.64	9.06	60.71
4	98-01-1	Furfural	0.25	/	0.99	/	1.73	/	100.00	/	66.82
5	2548-87-0	2-Octenal, (E)-	0.014	/	/	/	/	/	80.01	/	/
6	57266-86-1	(Z)-2-Heptenal	0.056	/	/	/	/	28.39	/	/	/
7	2305-05-7	ç-Dodecalactone	0.007	/	/	/	/	/	44.83	/	/
8	108-50-9	2,6-Dimethyl-pyrazine	0.25	/	0.23	/	0.64	/	24.46	/	60.80
9	14667-55-1	Trimethyl-pyrazine	0.19	/	0.15	/	0.53	/	16.86	/	37.62
10	90-05-1	2-Methoxy-phenol	0.04	/	/	/	/	/	54.88	/	/

Note: / means not detected. Odor thresholds for volatile substances were obtained from Compilations of odour threshold values in air, water and other media (second enlarged and revised edition).
